# The Elemental Fingerprints of Different Types of Whisky as Determined by ICP-OES and ICP-MS Techniques in Relation to Their Type, Age, and Origin

**DOI:** 10.3390/foods11111616

**Published:** 2022-05-30

**Authors:** Magdalena Gajek, Aleksandra Pawlaczyk, Krzysztof Jóźwik, Małgorzata Iwona Szynkowska-Jóźwik

**Affiliations:** 1 Faculty of Chemistry, Institute of General and Ecological Chemistry, Lodz University of Technology, Zeromskiego 116, 90-543 Lodz, Poland; aleksandra.pawlaczyk@p.lodz.pl (A.P.); malgorzata.szynkowska@p.lodz.pl (M.I.S.-J.); 2 Faculty of Mechanical Engineering, Institute of Turbomachinery, Lodz University of Technology, Wolczanska 217/221, 93-005 Lodz, Poland; krzysztof.jozwik@p.lodz.pl

**Keywords:** whisky, elemental analysis, ICP-MS, ICP-OES, CV-AAS, spirits, PCA, metals

## Abstract

A total of 170 samples of whisky from 11 countries were analysed in terms of their elemental profiles. The levels of 31 elements were determined by Inductively Coupled Plasma Mass Spectrometry (ICP-MS): Ag, Al, B, Ba, Be, Bi, Cd, Co, Cr, Cu, Li, Mn, Mo, Ni, Pb, Sb, Sn, Sr, Te, Tl, U, and V, Inductively Coupled Plasma Optical Emission Spectrometry (ICP-OES) Ca, Fe, K, Mg, P, S, Ti, and Zn and Cold Vapor-Atomic Absorption (CV-AAS): Hg techniques in those alcoholic samples. A comparative analysis of elemental profiles was made on the basis of the content of chosen elements with regard to selected parameters: country of origin, type of whisky (single malt and blended) and age of products. One of the elements which clearly distinguishes single malt and blended types of whisky is copper. Single malt Scotch whisky had a uniform concentration of copper, which is significantly higher for all malt whisky samples when compared with the blended type. Analysis of samples from the USA (n = 26) and Ireland (n = 15) clearly revealed that the objects represented by the same product but originating from independent bottles (e.g., JB, JDG, BUS brands) show common elemental profiles. On the other hand, comparative analysis of Scotch whisky with respect to aging time revealed that the longer the alcohol was aged, (i.e., the longer it stayed in the barrel), the higher the content of Cu and Mn that was recorded.

## 1. Introduction

Whisky (whiskey—alternate spelling is commonly used in Ireland and the USA—for consistency, the former spelling is used in this paper) is one of the most popular high-percentage alcoholic beverages made from grain in the world. In accordance with the present definition, whisky is a kind of distilled spirit made from fermented grain mash. Many types of whisky are associated with various types of production. In the case of European products, the alcohol should be matured for at least 3 years in wooden barrels of a volume not exceeding 700 L, and only water and caramel (for colouring) can be added to the distillate. For example, similar requirements for the type of grain from which whisky is produced are applied to both Scottish and Irish beverages. However, in Scotland, double distillation is used, while in Ireland it is tripled. The possibility for adding exogenous amylolytic enzymes in the mashing process for Irish whisky is another difference. In turn, alcohol produced in the USA (American Bourbon) is most typically aged less than 4 years (e.g., 2 years for the European market). Furthermore, bourbon in the USA has to be produced from a mixture of grains consisting of no less than 51% corn. The distillate must contain no more than 80% pure alcohol. Moreover, maturation takes place in new oak barrels, fired from the inside, which significantly differentiates this process from the one used in the production of European whisky, which is matured in previously used barrels (after wine, bourbon, or beer maturation process). In the USA, the top producers of bourbon have their distilleries in Tennessee and Kentucky. Alcohol branded as “Tennessee whisky” is known to have been subjected to a 10-day purification process using a layer of charcoal prepared from maple wood [1,2,3].

The analysis of whisky, both in terms of chemical composition characterisation and authentication, mostly involves the employment of separation techniques, such as gas or liquid chromatography, often coupled with flame ionization detection (FID) or mass spectrometry (MS). It needs to be highlighted that these techniques are mostly applied as a target type of analysis, where specific markers are traced and determined within the whisky authenticity verification [4,5,6,7,8]. Apart from VOCs and other organic compounds that, determined by the chromatographic methods, can be used as indicators for the identification of origin, alcoholic beverages can be also tested for trace elements, which are derived from the raw materials, production process equipment, storage vessels, and additives. Compared with those on organic markers, studies on trace elements used for the identification of counterfeit whisky are very limited. The first attempts to determine metals in whisky samples were made in 1998. Anodic stripping voltammetry (ASV) and atomic absorption spectroscopy (AAS) were applied at that time to measure and compare the levels of Zn, Pb, and Cu in four whisky samples. The authors noted that the stripping method had an important advantage over AAS in terms of lower detection limits. In the case of ASV, these limits were 4, 18, and 100 times lower for Zn, Cu, and Pb respectively. This is a key problem for heavy metals (Pb) since it is impossible to measure them using the AAS technique [9]. In 2002, Adam et al. [10] conducted trace elemental analysis on 35 Scotch whisky samples to verify whether there were trace element fingerprints characteristic of different kinds of Scotch whisky. A total of 31 samples of single malts, 1 sample of malt blend, 2 samples of blended Scotch, and 1 sample of grain whisky were analysed. For only the measurement of copper, an additional number of whisky samples was studied (6 blended Scotch whiskies, 11 single malt whiskies, and 1 rye whisky). The samples were taken directly from whisky bottles purchased from a supermarket. The selected malts originated from 4 Scottish regions: the Lowlands, the Highlands, Speyside, and Islay, and were aged between 6 and 20 years. For the determination of the selected metals, a graphite furnace atomic absorption spectrometer (GFAAS) was employed. The authors of the paper stated that the fingerprint of the metal concentrations in whisky could not be used as a criterion to identify whiskies from different production regions. However, when the second set of samples (42 malt whiskies and 8 blended whiskies) was analysed for copper, the concentration of this element could have been, according to authors, used as a criterion to distinguish blended or grain Scotch whiskies from malt whiskies. Much higher levels were observed in the malt whiskies in comparison with the concentration of copper in the blended Scotch whiskies or the pure grain whiskies. The authors concluded that the difference between these levels was highly significant. It was suggested that the copper analysis itself could be used as one of the markers to distinguish between blended and single malt Scotch whiskies. The main sources for the presence of copper in whisky are the copper stills in which whisky is distilled and the barrels in which the spirits are aged. Additionally, the authors indicated a possible relationship between the copper content and the acidity of the alcohol. In 2017, Shand et al. [11] made an attempt to use the elemental analysis of Scotch whisky performed by total reflection X-ray fluorescence as a potential tool in the identification of counterfeits. Elements such as Cu, Zn, Fe, Ca, S, Cl, K, Mn, P, Rb, and Br were selected because their presence is associated with the whisky production process. Moreover, their concentrations in most samples were above the limits of detection offered by TXRF. In total, 32 samples were analysed, of which 17 were single malt whiskies produced in different regions of Scotland (the Highlands, the Lowlands, Speyside, and Islay), 8 samples were blended scotch whiskies, and 2 were grain whiskies. Additionally, 5 samples were counterfeit whiskies from sources which remained anonymous. The samples were analysed without any special preparation process. A total of 18 out of the 32 were also checked by ICP-OES (after earlier sample preparation by the evaporation to dryness and the addition of nitric acid). In order to discriminate between the whisky samples in accordance with the indicated parameters, the authors used multivariate analysis. The principal component analysis (PCA) indicated that the counterfeit samples could be distinguished from the others on the basis of their trace elemental profiles. The second component was especially important in separating the counterfeit samples from the authentic Scotch whiskies. In turn, the third component had the greatest impact on the separation of classes (Highland, Lowland, Speyside, Islay, blended, grain, and counterfeit). The authors also observed statistically significant and strong positive correlations between Rb, K, and Mn. However, there was no obvious chemical or geochemical connection between these elements, which was underlined by the authors. Additionally, the applied CA analysis showed the unambiguous grouping of counterfeit samples. The linear discriminant analysis (LDA) made it possible in most cases to correctly classify the studied whisky samples into appropriate groups. It was extremely important, especially for the counterfeit samples.

Due to the fact that only a few, limited papers on metal analysis of whisky are available, the main goal of the authors was to perform an extensive, elemental characterization of whisky samples. Moreover, the possibility of using statistical analysis and chemometric tests to differentiate and distinguish whisky samples, based on their origins, types, and ages, was tested. Therefore, the present work presents an extremely rare approach to the assessment of selected whisky parameters, based on extensive elemental analysis. It should be emphasized that, in this study, a wide range of measurements was carried out with the use of 3 analytical techniques (ICP-MS, ICP-OES, and CV-AAS) to determine the concentrations of 31 elements in 170 whisky samples.

## 2. Materials and Methods

### 2.1. Samples

In total, 170 whisky samples (152 various brands of single malt and blended, high-percentage alcoholic beverages) originating from 11 countries (Scotland, the USA, Ireland, Poland, Japan, the United Kingdom, India, Azerbaijan, Slovakia, Wales, and Bulgaria) were chosen for elemental analysis using the by Inductively Coupled Plasma Mass Spectrometry (ICP-MS), Inductively Coupled Plasma Optical Emission Spectrometry (ICP-OES), and Cold Vapor-Atomic Absorption (CV-AAS) techniques. Alcohol samples selected in this study consisted partially of brands of whisky widely available on the Polish market, which can be found in the supermarkets. Some of the samples were obtained through official whisky distributors. Some of them are distillates intended for concentration and are thus not available for direct sale. The names of the whiskies are coded, and the manufacturers’ names are not given in this paper. The basic characteristics of the tested samples are included in Table 1.

### 2.2. Samples Preparation and Equipment

- ICP-OES and ICP-MS

The following measurement techniques were applied in this study: ICP-OES (Thermo Scientific, ICAP 7000 series, Bremen, Germany) and ICP-MS (Thermo Electron Corporation, X SERIES, East Lyme, CT, USA). These techniques required that the samples be prepared in a decomposed form, which process was performed in a microwave system (Ultrawave system, Milestone, Via Fatebenefratelli, Italy). For this purpose, 4 mL of 69–70% HNO_3_ (Baker, Avantor Performance Materials Poland S.A., Gliwice, Poland) were added to 4 mL of each of the samples. The acid was added in small portions due to the strongly exothermic nature of the reaction. In the next step, microwave mineralization was employed. The procedure was analogous to that used on the whisky samples described in our 2019 preliminary study [12]. After the mineralization process, the contents of the tubes were quantitatively transferred to flasks with a volume of 25 mL. A standard of In with a certified concentration was used as an internal standard to monitor signal stability (Merck, Warszawa, Poland). For the measurement of the indicated elements, it was necessary to prepare calibration curves based on a standard solution of CPAchem (Multi-element ICP standard, Stara Zagora, Bulgaria), and some single-element standards of In (ICP class, Merck, Darmstadt, Germany), Sb (ICP class, Merck, Darmstadt, Germany), Sn (ICP class, Chem Lab NV, Zedelgem, Belgium), Ti (ICP class, Radian International LLC, Austin, TX, USA), S (ICP class, Merck, Darmstadt, Germany), and P (ICP class, SCP Science, Québec, Canada). The preparation of the standards was carried out by the subsequent dilution method. The blank samples were prepared in the same way as the studied samples. 

An ICP-MS analytical technique was applied to determine the levels of metals in the whisky samples based on the following isotopes: ^107^Ag, ^27^Al, ^11^B, ^138^Ba, ^9^Be, ^209^Bi, ^111^Cd, ^59^Co, ^52^Cr, ^63^Cu, ^7^Li, ^55^Mn, ^95^Mo, ^60^Ni, ^208^Pb, ^121^Sb, ^118^Sn, ^88^Sr, ^125^Te, ^203^Tl, ^238^U, and ^51^V. In turn, concentrations of Ca (393.366 nm), Fe (238.204 nm), K (766.490 nm), Mg (279.553 nm), P (185.942 nm), S (180.731 nm), Ti (334.941 nm), and Zn (213.856 nm) were determined by the ICP-OES technique. Information about the operating conditions for the elemental analysis of the whisky samples performed using the ICP-OES and ICP-MS spectrometers is presented in Table S1 in the Supplementary Materials.

A total of three replicates were made for each alcohol beverage sample and analytical technique. The RSD, expressed as a percentage, even for elements measured at very low levels, was in the range of 0.01–5.00%. The accuracy of the applied procedure was verified based on the analysis of the certified reference material of TMDA 54.6 (a fortified lake-water sample from the National Water Research Institute, Burlington, Halton, ON, Canada). The obtained recoveries were close to 100%. The same procedure for verifying the accuracy of the proposed method was described previously by Gajek et al. in 2021 [13].

The coefficient of linear regression for each analyte was in the range from 0.999 to 1.000. The sensitivity of the developed method was considered in terms of the limit of detection (LOD) and the limit of quantification (LOQ). The two limits were based on values of the standard deviation of the results obtained for a series of blank samples, according to the following mathematical expressions: LOD = x_śr_ ∙ 3SD and LOQ = 3 ∙ LOD [14]. The obtained results are presented in Table S2 in the Supplementary Materials.

- CV-AAS

In this study, an automatic mercury analyser MA-3000 (Nippon Instruments Corporation, Tokyo, Japan) was applied to determine the total mercury content in the whisky samples. The analytical procedure was analogous to the one described in detail in 2019 [12].

### 2.3. Data Analysis

Statistica 12.5 (New York, NY, USA) software was used for the statistical and multivariate analysis. In order to verify the normality of the distribution of the studied variables, Kołmogorow–Smirnow tests were used. Kołmogorow–Smirnow tests are very helpful in the verification process if a sample originates from a population with a specific distribution based on the distance between the empirical distribution function of the sample and the cumulative distribution function of the reference distribution. The application of these tests for the significance level α = 0.05 showed that the hypothesis of a normal distribution for all analysed variables (the concentrations of 30 elements) should be rejected. For this reason, the Kruskal–Wallis non-parametric test was used to assess the significance of differences in the determined levels of elements among particular groups according to the parameters considered, such as country of origin, type, and year. The test determines whether the medians of two or more groups are different. The quantitative data were expressed in this study in the form of the box and whisker plots with a median value chosen as a central value. A total of 50% of the most common results are within the box, while the whiskers are limited by the highest and lowest results obtained in this work. To increase the interpretability of the results, multivariate analysis, namely principal component analysis (PCA), was applied. PCA is the basis of multivariate data analysis based on projection methods. The most important application of PCA is to represent multivariate data as a smaller set of variables in order to observe trends, clusters, and outliers. This analysis may uncover the relationships between observations and variables, and among variables themselves.

## 3. Results and Discussion

### 3.1. Levels of Metals in Analysed Whisky Samples

In this study, the levels of 31 elements in 170 whisky samples were determined. The concentrations of Ag, Al, B, Ba, Be, Bi, Cd, Co, Cr, Cu, Li, Mn, Mo, Ni, Pb, Sb, Sn, Sr, Te, Tl, U, and V were measured by the ICP-MS technique, but to assess the level of Ca, Fe, K, Mg, P, S, Ti, and Zn, the ICP-OES technique was used. The Hg content was analysed by the CV-AAS technique. In the collected data set, some of the obtained results were below the quantification limits. Te was not quantified in the majority of samples—157. Ag was not detected in 102 samples, nor Sb in 90, nor Ti in 61. Fe was not detected in 53 samples, Zn in 48, P in 34, V in 33, nor Mo in 31 samples. Cd was not determined in 20 samples, Sn in 11, Bi in 10, Tl in 9, nor K in 8 samples. U and Al were not detected in 5 independent samples, B in 2, and neither Pb nor Be was quantified in 1 sample. In the case of mercury content, all results were below the limit of quantification. Thus, this element was excluded from further calculations.

The first step in the work of data processing was to check the hypothesis about the type of distribution. For this purpose, the Kołmogorow–Smirnow test was used to verify the distribution of all analysed samples at the accepted level of significance, *p* = 0.05. The null hypothesis regarding the normal distribution for all variables was rejected. Therefore, the nonparametric Kruskal–Wallis test was used to further analyse the data. The basic statistical information on the studied variables such as the mean, median, minimum, and maximum, has been placed in Table 2.

Despite the quality control of food products prior to their introduction into the market, both reports in the literature and earlier research conducted by authors of this paper [13,15] clearly indicate that the permissible standards can be exceeded. Based on the results obtained for low-percentage alcoholic beverages, such as wines or ciders, cases where both the international and national standards have been exceeded can be found in the literature. The results obtained for 180 samples of wine studied by Gajek et al., 2021 [15], revealed that, in the case of 18 wine samples, the maximum levels of some metals (Cd—8 samples, Pb—9 samples, and Cu—1 sample) were slightly exceeded according to the OIV standards [16]. A similar observation was found in a study by Woldemariam et al., 2011 [17], where, especially in the case of lead, significant exceedances in wines (from the Czech Republic—max content 1253 µg/L) were reported. On the other hand, in the case of the analysis of cider samples [13], the authors emphasized that, for elements such as Cd and Pb, the maximum obtained results exceeded only the standards for drinking water [18]. The standards for alcoholic beverages were maintained for both elements. 

In terms of the elemental whisky analysis, none of the authors dealing with this topic verified the potentially negative impact of exceeding the permissible maximum levels of the selected metals. The authors of this paper referred only to the internal national standards that define the maximum permissible content of the selected metals (Cd and Pb) in high-percentage alcohols [19]. The maximum lead content was set at 0.3 mg/L, and the maximum cadmium content at 0.03 mg/L. In this study, the permissible Pb level was exceeded only for 1 out of 170 analysed whisky samples (the blended whisky from Ireland—max content 450.9 µg/L). The limit value for Cd was not exceeded in any case. 

### 3.2. Elemental Analysis for Country of Origin

#### 3.2.1. General Characteristics

So far, numerous attempts have been made to correlate the chemical composition of whisky with its origin. Most scientists have used chromatographic (GC and HPLC) and spectrophotometric (UV–Vis) techniques along with complex mathematical models to assess the correlation between an alcohol’s composition and its geographical origin. In some cases, authors have stated that the conducted measurements did not provide sufficient information to distinguish among whisky samples with regard to their countries of origin [20]. On the other hand, other literature reports clearly suggest that it is possible to distinguish Irish whisky from Scotch and bourbon on the basis of a few markers determined by chromatographic techniques [21]. Other researchers, having only 11 samples of whisky of various origin, were able to discriminate amongst all 5 of the alcohol groups under consideration using the Headspace mass spectrometry technique [22]. So far, there are single scientific studies in which researchers have made an attempt to distinguish whisky origin using multi-elemental analysis. Adam et al., 2002 [10], stated that the fingerprint of the metal concentration of whisky cannot be used as a criterion for identifying whiskies from different Scottish production regions. The authors of this paper also presented similar considerations in their preliminary research [12]. In the 20 tested samples of whisky, originating from different countries (Scotland, the USA, and Ireland), no statistically significant differences were found in any of the cases although the authors indicated that some isotopes (^48^Ti, ^138^Ba, ^66^Zn, ^90^Zr, and ^118^Sn) created the characteristic “fingerprint” of the Irish-made whisky sample. On the other hand, the copper content based on isotope ^63^Cu was considered as crucial in distinguishing the type of whisky (single malt and blended).Additionally, it turned out to be impossible, based on the collected outcomes, to distinguish between various production regions of Scotland (the Lowlands, the Highlands, Speyside, and Islay). However, as the authors emphasize, the number of analysed samples could be too small for the aforementioned comparison to be carried out correctly.

On the basis of the Kruskal–Wallis test, the existence of statistically significant differences in the concentration of the following elements was demonstrated: Ag, Be, Bi, Ca, Cd, Cu, Fe, Li, Mn, P, Sb, Sn, Ti, V, and Zn (Table 3). In the case of all the mentioned elements, the existence of statistically significant differences was confirmed based on the level of significance (*p*), which was less than 0.05. The most important statistical information connected with the division of samples by country of origin is included in Table S3 and Figure S1A–P (Supplementary Materials). What should be highlighted is the fact that, for most of the elements for which the existence of statistically significant differences were confirmed, whisky originating from Scotland was listed in the majority of comparisons. Although whisky samples from Scotland were represented by the largest number of samples (106), only in the case of Cu and Cd were the highest median values were observed for these elements among all other studied groups. In turn, with the exception of Cu, Cd, Sn, Bi, and Ca, for the rest of the mentioned elements (Li, Be, V, Mn, Ag, Sb, Zn, P, Fe, and Ti) the whisky from the USA was characterized by the highest median values when compared with samples from other countries. However, in most comparisons, copper was one of the crucial elements presented.

Considering the order of concentrations of the studied elements for which statistically significant differences were confirmed, only for the selected metals the same tendencies can be observed; for example, for Fe and Ti, the following order for median values can be noted: OTH > PL > SCT > IRL > USA. For Zn and V, on the other hand, the order was as follows: SCT > OTH > IRL > PL > USA. In general, for elements such as V, Sn, Zn, Sb, and P, the lowest values were determined in samples from Scotland, while the highest ones were found for products from the USA. Not surprisingly, the lowest content of Cu was characteristic for the whisky from the USA, whereas the alcohol from Scotland had the highest level of this element. For the rest of the studied elements, no common order in relation to the country of production can be indicated. The observed differences only prove that samples from various countries have completely different elemental fingerprints.

#### 3.2.2. Characteristics of Samples from the USA

**Figure 1:**
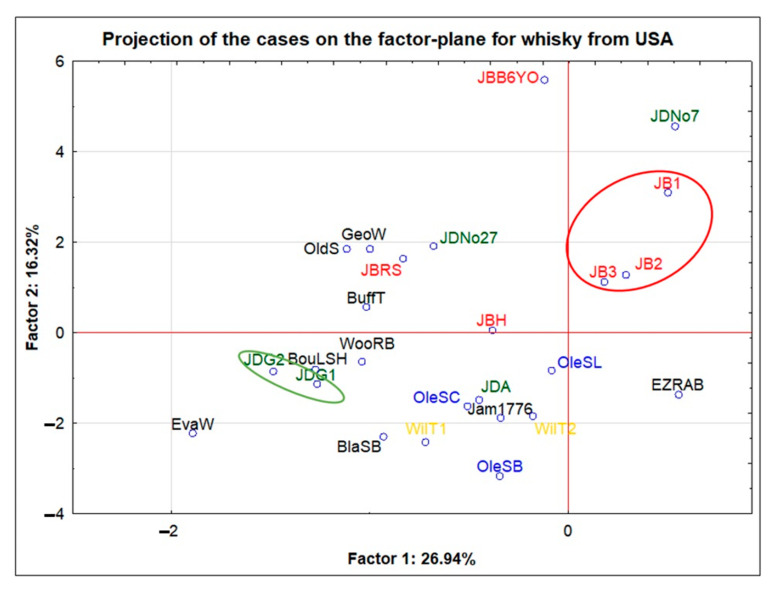
Projection of the cases on the factor-plane for 25 samples (after scale change) from the USA.

In the next steps, the samples originating from different countries will be discussed separately (USA—United States of America; IRL—Ireland; PL—Poland; OTH—other countries). The research objects from the USA consisted of 26 samples (each of the samples was coded.) Almost all of the samples from the USA were blended products only one of the tested samples was the single-barrel type of bourbon—BlaSB). Within this group, 12 independent brands were distinguished. The most numerous were the samples of the JB brand, which included 6 products (where JB1, JB2, and JB3 were samples of the same product, coming from different bottles, purchased in different stores during some period of time). The JD brand, which included 5 products, was also represented by a quite large group (where JDG1 and JDG2 were samples of the same product, coming from different bottles, purchased in different stores during some period of time). Moreover, only one of the studied group of samples was a product with an age declaration (JBB6YO). The remaining products were aged for the minimum period of time required by law. The projection of cases on the factor-plane which was made for this group clearly revealed one outlier point (JDS, from the JD brand). This was a limited-edition sample of a well-known brand of whisky from the USA. It should be emphasized that this sample was characterized by the highest content of the following elements in relation to the group under consideration: Li, Co, Ni, Cd, Sn, Tl, Bi, Zn, and P. In order to improve the readability of the graph and obtain a more accurate scale, this point was omitted from Figure 1.

The JDG1 and JDG2 (samples from independent bottles of JDG—marked in green) objects from the same brand, JD, in the projection shown in Figure 1, were placed next to each other. However, the remaining JD brand objects (namely JDA, JDNo7, and JDNo27) were dispersed, and therefore, no common elemental features were observed for them.

Objects JB1, JB2, and JB3, being the same product but coming from independent bottles (JB brand—marked in red), also very clearly created a common group in the presented projection. Objects JB2 and JB3 were very close to each other, while object JB1 was slightly shifted. This was probably due to the fact that the JB1 sample came from a bottle from a completely different production batch (the oldest one from the point of view of the purchase date). In relation to the group of objects JB1-3, the other JB brand objects were outlier points. They differed in their aging period in the barrel (JB6YO) or in the addition of flavouring substances (JBH and JBRS). 

Although, in the case of the remaining brands represented by more than one sample (e.g., Ole and WilT), it cannot be concluded that they formed common clusters characteristic of the brands, it should be emphasized that these samples were located in one quadrant (III) of the projection of cases on the factor-plane.

Additionally, the existence of statistically significant differences within the whisky samples from the USA was checked by the non-parametric test. The samples were divided according to the producers. Five groups were distinguished (JB, JD, Ole, WilT, and Oth, with the Oth group containing the rest of the single objects). The existence of statistically significant differences was revealed for Mg, Mn, Mo, Pb, and U. Among the mentioned elements, no statistically significant differences were found for Cu contents since the level of this metal was the lowest in samples originating from the USA. For magnesium, the differences concerned the JD and Ole brands, as well as the JD brand and other samples. Similarly, in the case of manganese, differences were noted for the JD brand and the group of other samples. For molybdenum, the differences concerned the JB and Ole brands, as well as the JB brand and other samples. For lead, a difference was found between the JD and Ole brands, and for uranium between the JB and Ole brands. It should be noted that the JD brand was distinguished by the lowest values of Mg and Mn in relation to the other groups. On the other hand, the JB brand was characterized by the highest Mo and U contents in relation to the others. The most important statistical information connected to the division of samples against the brands produced in the USA is included in the Supplementary Materials (Table S4).

#### 3.2.3. Characteristics of Samples from Ireland

**Figure 2:**
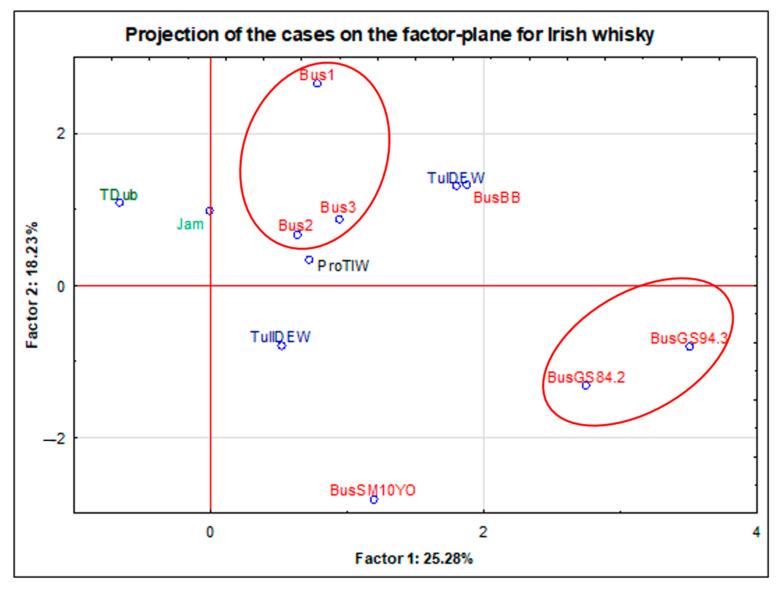
Projection of the cases on the factor-plane for 12 samples (after scale change) from Ireland.

Samples of whisky from Ireland included 15 subjects. Within this group, 6 independent brands were distinguished. The most numerous were the samples of the Bus brand (where Bus1, Bus2, and Bus3 were samples of the same product, coming from different bottles, purchased in different stores during some period of time). The projection of cases on the factor-plane which was made for this group clearly revealed three outlier points. Two of them belonged to the Jam brand (each of these alcohols was aged in a different barrel.) The last outlier is Southern Ireland’s blended whisky. The drink is a combination of 4-year-old barley distillates with 3-year-old grain distillates. Again, in order to improve the readability of the graph, these points were omitted from Figure 2.

The conducted projection revealed that the objects derived from the same product (Bus1, Bus2, Bus3—marked in red) have a uniform elemental profile, thanks to which, they form a common group. These samples were characterized by the highest content of barium compared to the other samples from Ireland. As with the products from the USA, objects Bus2 and Bus3 were very close to each other, while object Bus1 was slightly shifted. This was probably due to the fact that the JB1 sample came from a bottle from a completely different production batch (and oldest in terms of time of its purchase). BusGS84.2 and BusGS94.3 (marked in red) were samples of single-grain distillates from the Bus brand. These distillates were high-percentage alcohols without an aging process. These were unique samples obtained directly from the distillery, provided by one of the Polish distributors. Again, it can be concluded that the samples of these alcohols had a very consistent elemental profile. They were characterized by a higher content of copper, chromium, and nickel than the others. The other 2 samples from the Bus brand were the 10-year-old single malt (Bus10YO) and the premium-class blend consisting of 75% malt whisky (BusBB). The 10-year-old single malt (Bus10YO) sample seemed to be particularly interesting in terms of elemental composition. It was characterized by the highest values in relation to the other samples from Ireland in terms of the following elements: V, Mn, Ni, Cu, Sr, Sn, and P. As in the case of the JB brand from the USA, the sample with the declared aging period was clearly the outlier. Similarly, in this case, the BusSM10YO object was the only one of the Bus brand products with a declared aging period. This suggests that time may be the most important distinguishing factor (as opposed to brand or origin).

Moreover, as in the case of the product from the USA, the samples from Ireland were divided according to the producers (Bus, Jam, Tul, and Oth: the rest of the single objects). On the basis of the Kruskal–Wallis test, the existence of statistically significant differences in the concentration of B was demonstrated between the group of the products of the Jam and Bus brands. The Jam brand was characterized by a higher content of this element. The most important statistical information for B is included in the Supplementary Materials (Table S5).

#### 3.2.4. Characteristics of Samples from Poland

The set of whisky samples from Poland contained 10 objects, including 8 different brands. Poland is certainly not a country associated with whisky production. However, in recent years, due to rapidly growing consumption, products from domestic brands have been appearing on the market. Most often, producers of vodkas, liqueurs, or mead, wanting to expand their product range, have introduced whisky produced from local ingredients to their portfolios. There are also beverages on the Polish market that are advertised as Polish products but created in cooperation with manufacturers from other countries (most often Scotland). Frequently, they are blended types of whisky made of Scottish barley malts and Polish distillates from other cereals.

**Figure 3:**
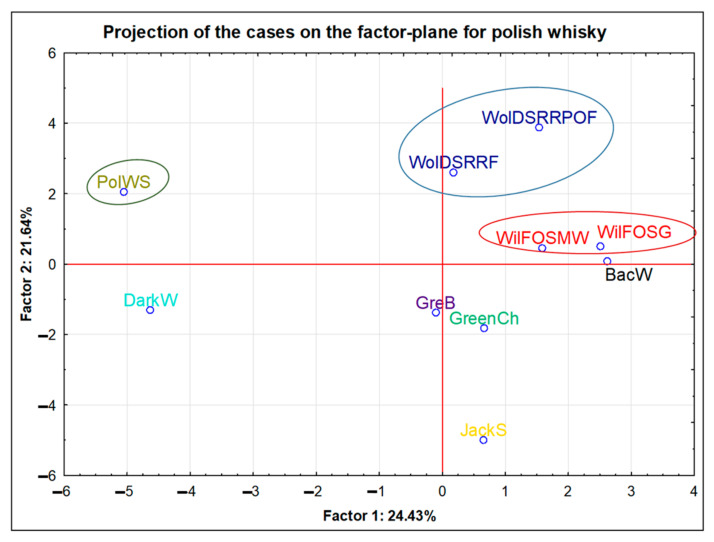
Projection of the cases on the factor-plane for the 10 samples from Poland investigated in this study.

The conducted projection of the cases on the factor-plane revealed that the samples from the same manufacturers (WolDS and WilFO) were grouped together (Figure 3). In the case of samples from the WilFO distillery (marked in red), one was a single-grain (WilFOSG), and the second was a single-malt wheat (WilFOSMW). However, the same production method and distillation equipment ensured, in this case, a coherent elemental profile. Moreover, both products from this brand were aged for a period of 3 years. Characteristic features of the WilFO brand were the highest Sn and Pb contents compared to other products from Poland. In turn, samples from the WolDS distillery (marked in navy blue) are single rye whiskies. The main difference between them is the type of barrel in which they were matured. The sample of WolDSRRF was aged in rum barrels, whereas the whisky coded as WolDSRRPOF was matured in a barrel made of Polish oak. As in the case of the previous brand, the maturation period was 3 years. It should be emphasized that the WolDS brand is distinguished from the others due to its having the highest values of Mn and Mg.

An interesting position in the compared group of samples was PolWS (a Polish single malt whisky—marked in dark green). It was produced at home, but according to the definition, it met all the requirements for this type of alcohol. This whisky was aged for 3 years in oak barrels, fired from the inside. What distinguished this sample was its having the highest content of Sr, K, S, and P compared to the other Polish products. Potassium was indicated by Gajek et al., 2021 [13], as an element which much greater content characterizes home-made products.

Among the 10 analysed samples from Poland, 5 were single malt, single rye, or single grain and were located in the projection of the cases on the factor-plane at the top of the plot (quarters I and II). The remaining 5 samples were blended-type products. These points were at the bottom of the projection (quarters III and IV). However, in the case of all 5 blended samples, despite the fact that the manufacturer declared the Polish origin of these products, it was extremely difficult to trace them back to their real origin.

#### 3.2.5. Characteristics of Samples from Scotland

The group of products from Scotland included 106 whisky samples (50 single malt and 56 blended whiskies). The studied objects in this group of products were extremely diverse in terms of price. They included both low-end products, commonly available in supermarkets, and high-quality, single malt whiskies, including items not available for commercial sale. In such a diverse group of samples, making comparisons analogous to those we made for the samples from Ireland, the USA, or Poland, taking into account the manufacturer, was extremely difficult (more than 60 independent producers were investigated.) There were no statistically significant differences in any case in reference to the brands. Due to the fact that the group of samples from Scotland was much more diversified than those from other countries, the influence of additional parameters, such as the type and age of alcohol, on the grouping of objects was taken into account. Therefore, in the next steps, we verified the hypothesis about the influence of the type of Scotch whisky (blended or single malt) and the aging time (divided into 3 groups based on maturation period) of the single malt whisky from Scotland on the ability to distinguish samples.

### 3.3. Elemental Analysis for the Type of Scotch Whisky

In order to verify the differences in the types of whisky, namely single malt and blended, only products originating from Scotland were taken into account. Therefore, 106 samples were analysed, including 50 single malt whiskies and 56 blended whiskies. The non-parametric test showed the presence of statistically significant differences between the content of such elements as: Al, Cr, Cu, Fe, K, Mg, Mn, P, S, Ti, Tl, Zn, and V (Table S6 and Figure S2A–K). Taking into account the median value for the following elements, in this group, a blended whisky contained more Al, Cr, and Tl when compared with single malt whisky. In turn, for the rest of the elements (Cu, Fe, K, Mg, Mn, P, S, Zn, and V), higher amounts were observed in single malt whisky. Additionally, a projection of the cases on the factor-plane for all samples originating from Scotland was carried out. As shown in the graph, most of the single malt whisky samples were grouped on the left side. In turn, a vast majority of blended whisky samples was placed on the right side of the projection of the cases plot (Figure S3). The outlier point (marked in orange) is a 26-year-old, extremely rare, high-quality single malt whisky, which was not commercially available, and was characterized by an increased content of the following elements: Mn, Co, Cu, Mg, K, and P. The second outlier point (marked in green) was the sample of single malt whisky stored in special, small barrels (octave barrels). Aging the alcohol in much smaller barrels of about 65L will ensure a better integrity of the beverage with the wood. Despite the short maturation period (3 years), the alcohol is much more “saturated” and richer in taste, (in the case of the present study, this sample was characterized by higher levels of the following elements compared to other samples from Scotland: Li, Co, Mo, Bi, and Zn.). In our preliminary studies [12], we performed a semi-quantitative measurement of the following 21 isotopes: ^44^Ca, ^45^Sc, ^47^Ti, ^48^Ti, ^51^V, ^52^Cr, ^54^Fe, ^55^Mn, ^60^Ni, ^63^Cu, ^66^Zn, ^88^Sr, ^90^Zr, ^95^Mo, ^101^Ru, ^107^Ag, ^111^Cd, ^118^Sn, ^138^Ba, ^208^Pb, ^209^Bi, and total Hg content using the ICP-ToF-MS and CV-AAS techniques. We tried to differentiate 20 whisky samples according to country of origin, production region of Scotland, and type of whisky (single malt and blended). The performed analysis revealed the existence of statistically important differences between single malt and blended whiskies for Cr, Fe, Cu, Zn, and Ba. The median counts for copper, chromium, and barium were higher for single malt whisky. In turn, for iron and zinc, the blended whisky samples were characterized by higher counts of the mentioned elements. In our previous study, the analysed samples from various Scottish production regions differed in age, type, and brand. Most of the single malt samples were matured whiskies, while the blended whiskies were mainly 3-year-old products. Unquestionably, this parameter (age) could have affected the existence of statistically significant differences in the whiskies’ contents of Cr, Fe, Cu, Zn, and Ba.

Admittedly, the projection of cases on the PCA plot, which was carried out in this study earlier, did not show any grouping by brand or production region. Nevertheless, the obtained PCA graphs potentially suggested a simultaneous overlapping of two parameters, such as age and type. Thus, in order to evaluate the influence of the type of whisky on the elemental compositions, the data set in this work was significantly reduced from 106 objects to 71. Only samples with the same aging period (3 years) were included in the new tested data set, which consisted of 54 blended whiskies and 17 single malt whiskies. In this case, statistically significant differences were reported for such elements as Al, Cr, Cu, Fe, K, Mg, Mn, S, Ti, Tl, and V. Therefore, in relation to the former comparison of all samples from Scotland (106 objects), no statistically significant differences for P and Zn were stated. This allowed us to conclude that these elements (P and Zn) could be related to the age parameter since the influence of this factor was theoretically eliminated as a consequence of the rejection of samples maturated for longer than 3 years.

**Figure 4:**
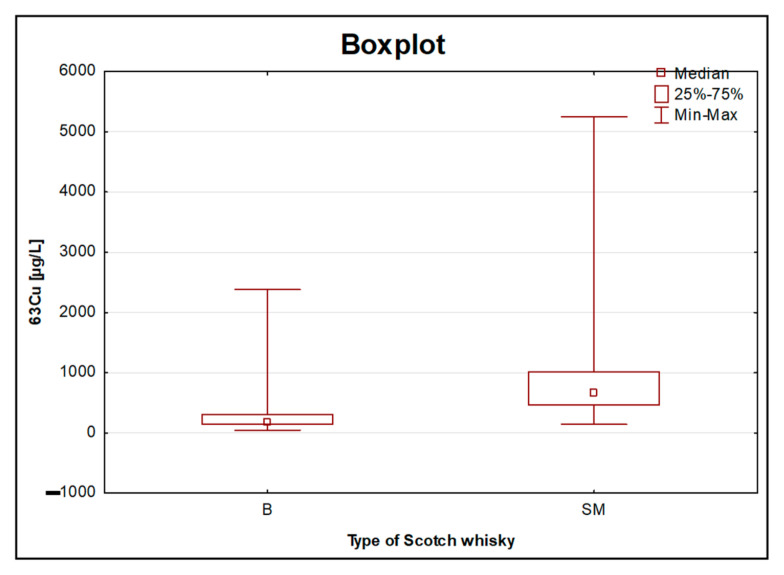
Boxplot for the concentration of Cu obtained for 106 objects of Scotch whisky, divided into two groups: blended (B) and single malt (SM).

**Figure 5:**
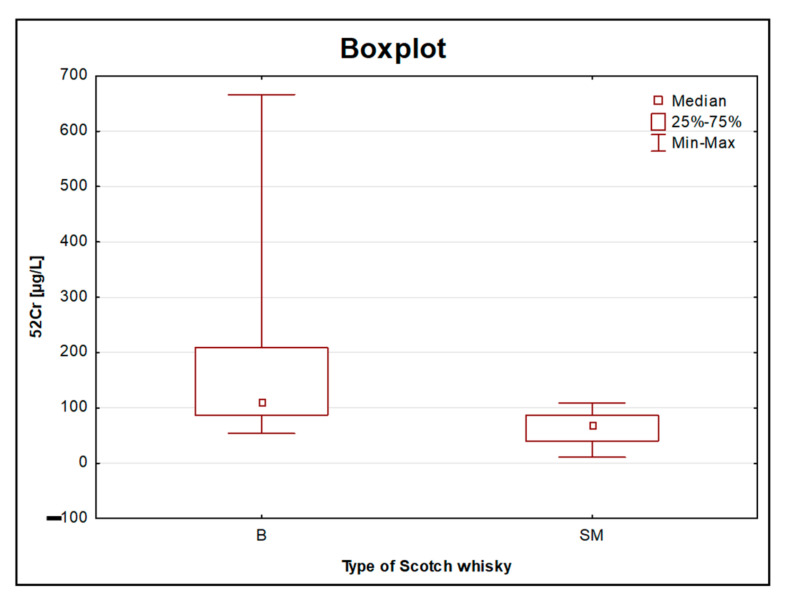
Boxplot for the concentration of Cr obtained for 106 objects of Scotch whisky, divided into two groups: blended (B) and single malt (SM).

As the authors of this article showed in the preliminary studies [12] conducted on a much smaller number of objects, the origin of the traces of Cu (Figure 4) could be alembic, which as a rule, is made of copper. This metal enters into a chemical reaction with a distillate and somehow “extracts” sulphuric aromas from it. Moreover, literature reports suggest that copper ions have such a profound effect on the flavour profile of all malt whiskies that has been described as the “fourth ingredient”, after malted barley, water, and yeast. Moreover, it was noted that systematic changes within the heating and cooling elements of pot stills can affect copper solubility and hence spirit character [23]. The size of the alembic is extremely important since the longer the distillate touches the copper elements, the softer it will be. Malt whisky is produced in traditional copper stills in batch-type rectors, while grain whisky, which in general contributes the most to the blended whisky, is run continuously using more industrial-style patent stills. Therefore, malt whisky, being distilled in small traditional pot stills, is naturally expected to contain more copper than other types of whisky produced during column still distillation. The sample with the highest Cu content (5252 [µg/L]) was the previously mentioned 26-year-old, single malt whisky. Adam et al., 2002, also confirmed that the whisky had a uniform copper concentration and that the mean copper concentration was significantly higher for all malt whisky samples than for grain and blended scotch whisky samples [10]. The aforementioned grain whisky (with the largest share in blended whisky, especially in the lower price range) is produced with column stills, which are made from stainless steel. This equipment comprises a tall column structure attached above a boiling kettle, and it is designed so as to attain purer vapours [24]. There are several types that are made only of stainless steel, but for the vast majority of them, the composition includes elements such as chromium and nickel. One of the few metals with higher levels in blended whisky was the already-mentioned Cr (from one of the stainless-steel components) (Figure 5). The results obtained for chromium in the blended type of whisky were in the range of 53.83–666.1 [µg/L], whereas for single malt whisky, this range was much narrower (10.70–108 [µg/L]). As mentioned in the introduction, the number of scientific papers on the elemental analysis of whisky, especially those considering its type, is very limited. However, so far, a great deal of work has focused on the analysis of volatile organic compounds eluted using chromatographic techniques [2]. The results show that Scotch grain whisky from a continuous column still distillation contains very few congeners (substances other than the desired type of alcohol, ethanol, produced during fermentation), while Scotch malt whisky produced via double pot still distillation is much richer in them. For the remaining examined elements, despite the lack of statistically significant differences, in most cases, higher concentrations were observed in the single malt whiskies compared to the blended whiskies. Thus, this supports the hypothesis that this type of whisky is richer in various ingredients. The presented results may prove that the equipment used in the alcohol distillation process may have a significant impact on the elemental profile of the final product. 

### 3.4. Elemental Analysis for Age of Single Malt Scotch Whisky

In order to verify the hypothesis on the potential impact of aging time on the elemental composition of whisky, from the considered set of samples, 50 objects (Scottish single malt) were selected, where the producer declared the age of the alcohol. The samples were divided into the following groups: 3–9YO; 10–16YO; >16YO (where, in group 3–9 YO, there were only aged products, the minimum period required by law, i.e., 3 years). Considering the tested set of samples in terms of the age of whisky, the existence of statistically significant differences based on Kruskal–Wallis tests only for the concentrations of Cu (3–9YO—10–16YO and 3–9YO—>16YO) and Mn (3–9YO—>16YO) were found (Table 4 and Figure S4A,B in Supplementary Materials). An upward trend was observed for both elements. This means that, the longer the alcohol was aged, i.e., the longer it stayed in the barrel, the higher the content of these elements that was recorded. The mentioned trend is visible when taking into account both the median values and the other basic statistics (minimum and maximum values). It should be emphasized that, in this study, high-quality, single malt Scotch whisky was considered. It was previously proved that this type of alcohol was characterized by a higher content of copper, as a direct consequence of the method of production. The increase in the copper content correlated with the extended aging period is certainly related to the fact that the products with longer aging periods analysed in this study were the leading brands produced in Scottish distilleries. Thus (in accordance with the manufacturers’ declarations), dedicated distillation stills with longer “necks” are often used by leading brands in order to ensure special taste qualities. For the rest of the elements determined in this study, again based on the median value in most cases (V, Cr, Ni, Sr, Sb, Bi, Zn, Mg, K, P), despite the lack of statistically significant differences, the same trend was observed as for Cu and Mn. In the previous whisky comparison (single malt and blended), it was concluded that the differentiation of the samples may have been influenced by several, overlapping parameters. Therefore, the authors decided to compare the results only within the group of samples with the same aging period (3 years). Despite the lack of statistically significant differences for the studied elements, the influence of aging on the increased concentrations of Zn and P was clearly visible. Both the mean and median values for these elements increased in the following order: “3–9YO” < “10–16YO” < “>16YO”.

As other authors have noted, the concentrations of oak-derived congeners in a given cask of whisky increase with maturation time. Moreover, it is possible to create a graph showing maturation congener concentrations against age. There are literature reports of using near-infrared reflectance (NIR) as a predictive tool for Canadian whisky aging. Natural ^14^C in atmospheric carbon dioxide is absorbed by metabolism into all plants, including the cereals used for whisky manufacture. Analysis of the ^14^C levels in ethanol concentrated from the whisky samples was used to estimate the year in which the cereal was grown and then to relate this year with the age of maturation [25]. Chromatographic analysis of selected acids and phenols in chosen samples of whisky (from 6 to 30 YO) conducted by Ng et al., 2000 [26], brought a similar conclusion. The authors stated that, in general, the samples of the oldest whisky contain the highest concentrations of the analysed compounds.

In this work, an interesting relationship between the sulphur concentration and the age of the analysed alcohol beverages samples was made and deserves attention. Sulphur volatile compounds generated during the production process of whisky, to a large degree, influence their quality [27]. On the basis of the research carried out so far, alkyl sulphides such as DMS, DMDS, and DMTS have been recognized as alcohol maturation markers [28,29]. It has been proven that their levels decreased during maturation. In our study, taking into account the median values of S, its levels clearly decreased with age, which undoubtedly had a positive effect on the quality of alcohol. Thus, in our work, the same relationships were proven as made by other authors regarding the S since the sulphur compound levels determined by chromatographic techniques.

Additionally, a projection of the cases on the factor-plane for 50 samples originating from Scotland with the producer’s declaration of the age of the alcohol was carried out. Exactly as in the case of the single malt and blended whisky graph, the division of the plot into two parts can be noticed. We can observe a strong tendency that “older” products are on its left side, while the “younger” ones are mostly on the right side of the PCA plot (Figure S5). As for the previous comparison (Figure S3), the same outliers can be identified (i.e., the sample of the unique, 26-year-old whisky marked in orange and the sample of the whisky aged in octave barrels marked in green). Thus, a conclusion can be drawn only about general trends regarding the position of individual samples in the presented projections of cases on the factor-plane, supported by the presence of statistically significant differences. However, it should be emphasized that many parameters affect the possibility of the potential differentiation of particular groups of samples from one another.

## 4. Conclusions

Taking into account the national standards defining the maximum permissible levels of Cd and Pb in high-percentage alcohol products, it was found that the permissible level was exceeded in the case of Pb for only one sample. The limit value for Cd was not exceeded in any case. For the set of Scotch whisky samples (n = 106), the existence of statistically significant differences was indicated for metals such as Al, Cr, Cu, Fe, K, Mg, Mn, P, S, Ti, Tl, Zn, and V between the groups of single malt and blended whiskies. Single malt Scotch whisky had a uniform concentration of copper, and the mean copper content was significantly higher for all malt whisky samples than for the blended type. The main source of Cu could be alembic, which, as a rule is made of copper. Moreover, the presented results suggested that the equipment used in the alcohol distillation process may have a significant impact on the elemental profile of the final product. 

The analysis of the samples from the USA and from Ireland (n = 26) clearly revealed that the objects that were the same product but originated from independent bottles (e.g., the JB, JDG, and Bus brands) showed similar elemental profiles. From the consumer’s point of view, the elemental characteristics of whisky entirely produced in Poland from local raw materials, including home-made products, may seem interesting. Alcohol produced at home can be characterized by the highest content of Sr, K, S, and P as compared to other products from Poland. In terms of the aging time of whisky, the existence of statistically significant differences based on Kruskal–Wallis tests of the concentrations of Cu (3–9YO—10–16YO and 3–9YO—>16YO) and Mn (3–9YO—>16YO) was observed. The conclusion is that the longer the alcohol was aged, i.e., the longer it stayed in the barrel, the higher the content of these elements that was recorded. Based on the comparison of three aging periods only for single malt Scotch whisky, it can be concluded that, despite the lack of statistically significant differences for Zn and P, the influence of aging on the increasing concentration of these elements was clearly visible. Both the mean and median values for these elements increased in the following order: “3–9YO” < “10–16YO” < “>16YO”. The study of reduced data set (from 106 to 71 samples) for both type of Scotch whisky samples (single malt and blended) also allowed to conclude that P and Zn could be related to the age parameter. The influence of this factor was theoretically eliminated as a consequence of the rejection of samples maturated for no longer than 3 years, no statistically significant differences for these elements were stated. In this study, it has also been proven that levels of sulphur decrease during maturation.

## Figures and Tables

**Table 1:** Characteristics of the tested set of samples.

N	Scotland	USA	Ireland	Poland	Others	
	Single Malt—50	Single Barrel—1	Single Malt—3	Single Malt—3	Japan—3	UK—1
	Blended—56	Blended—25	Blended—12	Blended—7	India—3	Azerbaijan—1
					Slovakia—3	Wales—1
					Bulgaria—1	
Total	106	26	15	10	13	

**Table 2:** Basic statistics for determined elements for all whisky samples (n = 170) [µg/L].

Element	n	Mean	Median	Min	Max	Element	n	Mean	Median	Min	Max
Ag	170	4.270	<LOQ	<LOQ	399.1	Sb	170	3.470	<LOQ	<LOQ	227.9
Al		117.7	113.3	<LOQ	399.7	Sn		9.800	4.670	<LOQ	44.50
B		4388	4116	<LOQ	12.89	Sr		47.18	45.81	15.84	119.2
Ba		188.7	182.4	38.68	950.9	Te		0.040	<LOQ	<LOQ	1.200
Be		0.100	0.090	<LOQ	0.300	Tl		0.110	0.040	<LOQ	2.600
Bi		1.310	0.870	<LOQ	19.80	U		0.260	0.230	<LOQ	0.900
Cd		1.260	0.720	<LOQ	16.00	V		2.210	0.960	<LOQ	57.30
Co		4.530	2.470	0.406	74.90	Ca		14.66	9185	723.8	175.35
Cr		153.4	111.1	10.70	666.1	Fe		166.6	88.03	<LOQ	1485
Cu		473.7	216.0	16.25	5252	K		18.50	12.45	<LOQ	149.30
Li		21.36	12.27	0.474	399.5	Mg		1487	1046	208.5	11,548
Mn		47.43	32.95	4.396	286.5	P		1637	313.7	<LOQ	30.11
Mo		1.790	1.070	<LOQ	32.30	S		7126	4648	296.3	69.91
N		24.01	12.96	3.201	301.3	Ti		25.68	12.72	<LOQ	288.3
Pb		15.82	10.61	<LOQ	450.9	Zn		1221	177.5	<LOQ	31,458

**Table 3:** Groups for which statistically significant differences were reported.

Statistically Significant Differences	Elements
SCT–USA	Li; Be; V; Cu; Ag; Sn; Sb; Zn; P
SCT–IRL	Mn; Cu; Cd
SCT–PL	Sn
SCT–OTH	Bi; Cu
USA–PL	Ca
USA–OTH	Bi; Fe; Ti; Cu
IRL–OTH	Cu

SCT—Scotland; USA—United States of America; IRL—Ireland; PL—Poland; OTH—other countries.

**Table 4:** Contents of selected elements (with statistically significant differences) in the measured Scottish Single Malt Whisky (n = 50) [µg/L].

	Age	n	Mean	Median	Min	Max	Std. Dev.
55Mn	3–9 YO	17	63.09	54.73	16.85	155.1	34.36
	10–16 YO	28	76.93	69.25	22.04	223.1	42.41
	>16 YO	5	133.7	94.17	73.37	260.1	76.67
63Cu	3–9 YO	17	558.2	543.0	143.2	1163	289.9
	10–16 YO	28	982.0	766.6	172.8	2536	606.4
	>16 YO	5	1809	836.7	663.9	5252	1950

## Data Availability

Not applicable.

## Supplementary Materials

The following supporting information can be downloaded at: www.mdpi.com/article/10.3390/foods11111616/s1, Figure S1A–P: Box & whisker plots of selected elements (with statistically significant differences) in the measured whisky samples (n = 170) [µg/L]; Figure S2A–K: Box & whisker plots of selected elements (with statistically significant differences) in the measured Scottish whisky (n = 106) [µg/L]; Figure S3: Projection of the cases on the factor-plane for 106 samples from Scotland according to their type (single malt (SM) and blended (B)); Figure S4A,B: Box & whisker plots of selected elements (with statistically significant differences) in the measured Scottish single malt whisky (n = 50) [µg/L]; Figure S5: Projection of the cases on the factor-plane for 50 samples of single malt whisky from Scotland, Table S1: ICP-MS (Thermo Electron Corporation, X SERIES, East Lyme, CT, USA) and ICP-OES (Thermo Scientific, ICAP 7000 series, Bremen, Germany) parameters and measurement conditions; Table S2: Basic validation parameters obtained for each analyte by using developed method (n, number of standards in three replicates, R^2^, coefficient of determination); Table S3: Contents of selected elements (with statistically significant differences) in the measured whisky samples (n = 170) [µg/L]; Table S4: Contents of selected elements (with statistically significant differences) in the measured samples from USA division against the brand (n = 26) [µg/L]; Table S5: Contents of B in the measured samples from Ireland division against the brand (n = 15) [µg/L]; Table S6: Contents of selected elements (with statistically significant differences) in the measured Scottish whisky (n = 106) [µg/L].

## Sample Availability

Samples are available from the authors.
