# Supplementary material for: The Elemental Fingerprints of Different Types of Whisky as Determined by ICP-OES and ICP-MS Techniques in Relation to Their Type, Age, and Origin
Source: Foods. 2022 May 30;11(11):1616. doi: 10.3390/foods11111616 (PMC9180757; doi:10.3390/foods11111616)
Supplement: Supplementary file 1 [file foods-11-01616-s001.zip › foods-1691783-supplementary.pdf]

foods MDPI

Table S1. ICP-MS (Thermo Electron Corporation, X SERIES, East Lyme, CT, USA) and ICP-OES (Thermo Scientific, ICAP 7000 series, Bremen, Germany) parameters and measurement conditions.

Parameter and accessories	ICP-OES	ICP-MS
Number of replicates	3	3
Carrier gas	Argon	Argon
Plasma gas flow rate [L·min−1]	12	10
Auxiliary gas flow rate [L·min−1]	0.5	0.76
Nebulization gas flow rate [L·min−1]	0.5	0.9
Torch	Quartz	Quartz
Nebulizer	Concentric quartz	Concentric quartz
Generator power [W]	1150	1317
Internal standard	In	In

Table S2. Basic validation parameters obtained for each analyte by using developed method (n, number of standards in three replicates, R^2, coefficient of determination).

Analyte	n	Equation	R^2	LOD [µg/L]	LOQ [µg/L]
Ag	7	$y = 85.67x + 11.36$	0.999	0.183	0.550
Al	7	$y = 49.76x + 125.91$	0.999	0.737	2.212
B	7	$y = 66.68x + 75.45$	0.999	0.368	1.105
Ba	7	$y = 245.09x + 69.55$	0.999	3.399	10.20
Be	7	$y = 10.53x + 1.82$	0.999	0.025	0.074
Bi	7	$y = 329.78x + 15.00$	0.999	0.216	0.648
Cd	7	$y = 20.66x + 0.45$	0.999	0.057	0.170
Co	7	$y = 103.62x + 5.9$	0.999	0.093	0.279
Cr	7	$y = 78.94x + 49.09$	0.999	1.848	5.542
Cu	7	$y = 28.56x + 4.55$	0.999	2.453	7.360
Li	7	$y = 32.47x + 1{,}82$	0.999	0.116	0.347
Mn	7	$y = 108.13x + 68.18$	0.999	0.932	2.796
Mo	7	$y = 30.54x + 0.45$	0.999	0.270	0.810
Ni	7	$y = 23.61x + 1.82$	0.999	0.948	2.844
Pb	7	$y = 207.5x + 7.27$	0.999	2.167	6.502
Sb	7	$y = 54.38x + 0.45$	0.999	0.566	1.698
Sn	7	$y = 62.67x + 4.09$	0.999	1.468	4.404
Sr	7	$y = 171.82x + 7.73$	0.999	4.080	12.24
Te	7	$y = 4.35x + 0.45$	0.999	0.008	0.024
U	7	$y = 431.42x + 0.0$	0.999	0.008	0.024
V	7	$y = 102.66x + 62.27$	0.999	0.068	0.204
Ca	6	$y = 73844x + 9320$	1.00	0.033	0.100
Fe	6	$y = 16219x + 108.6$	1.00	720.5	2162
K	6	$y = 186.7x + 146.5$	0.999	15.00	45.00
Mg	6	$y = 50432x + 421.8$	1.00	611.0	1833
P	3	$y = 282.6x + 0.381$	1.00	14.00	42.00
S	3	$y = 576.3x + 17.41$	1.00	26.00	78.00
Ti	3	$y = 70413 + 9.800$	1.00	598.6	1796
Tl	7	$y = 290.65 + 0.909$	0.99	4.000	12.00
Zn	6	$y = 22772 + 520.3$	1.00	31.00	93.00

Table S3. Contents of selected elements (with statistically significant differences) in the measured whisky samples (n = 170) [μg/L].

Element	Code of country	n	Mean	Median	Element	Code of country	n	Mean	Median
^{7}Li	SCT	106	15.58	10.60	^{121}Sb	SCT	106	3.660	< LOQ
	USA	26	44.29	24.97		USA	26	8.280	0.861
	IRL	15	22.62	15.67		IRL	15	0.310	0.114
	PL	10	23.15	21.98		PL	10	0.330	0.170
	OTH	13	19.96	13.00		OTH	13	2.629	0.067
^{9}Be	SCT	106	0.090	0.090	Zn 213.856	SCT	106	1065	143.3
	USA	26	0.120	0.118		USA	26	2122	571.6
	IRL	15	0.110	0.098		IRL	15	512.3	193.5
	PL	10	0.130	0.110		PL	10	985.4	153.6
	OTH	13	0.139	0.137		OTH	13	548.2	157.6
^{51}V	SCT	106	1.080	0.870	P 185.942	SCT	106	1347	183.0
	USA	26	7.910	2.400		USA	26	2311	913.2
	IRL	15	1.260	1.129		IRL	15	2393	597.4
	PL	10	1.890	1.100		PL	10	2210	803.0
	OTH	13	2.578	1.058		OTH	13	1199	344.6
^{55}Mn	SCT	106	49.60	34.79	209Bi	SCT	106	1.310	0.920
	USA	26	48.55	42.47		USA	26	1.660	0.949
	IRL	15	24.03	20.47		IRL	15	1.380	0.987
	PL	10	86.82	33.74		PL	10	1.290	0.600
	OTH	13	34.25	32.05		OTH	13	0.491	0.436
^{63}Cu	SCT	106	584.5	375.8	Fe 238.204	SCT	106	154.5	94.55
	USA	26	106.3	39.15		USA	26	248.3	176.5
	IRL	15	82.51	72.11		IRL	15	276.1	125.6
	PL	10	201.3	228.2		PL	10	89.00	52.37
	OTH	13	999.2	243.3		OTH	13	53.01	9.994
^{107}Ag	SCT	106	5.560	< LOQ	Ti 334.941	SCT	106	23.79	13.21
	USA	26	2.580	0.468		USA	26	52.91	28.14
	IRL	15	0.630	< LOQ		IRL	15	13.47	15.98
	PL	10	4.520	0.760		PL	10	24.33	< LOQ
	OTH	13	0.971	0.360		OTH	13	7.628	< LOQ
^{111}Cd	SCT	106	1.310	0.840	Ca 393.366	SCT	106	15540	10752
	USA	26	1.520	0.647		USA	26	11359	6286
	IRL	15	0.900	0.237		IRL	15	12068	8125
	PL	10	0.840	0.350		PL	10	23888	20929
	OTH	13	0.807	0.311		OTH	13	8687	6275
^{118}Sn	SCT	106	7.060	3.570					
	USA	26	16.08	8.745					
	IRL	15	11.52	6.803					
	PL	10	15.73	7.550					
	OTH	13	15.79	10.60					

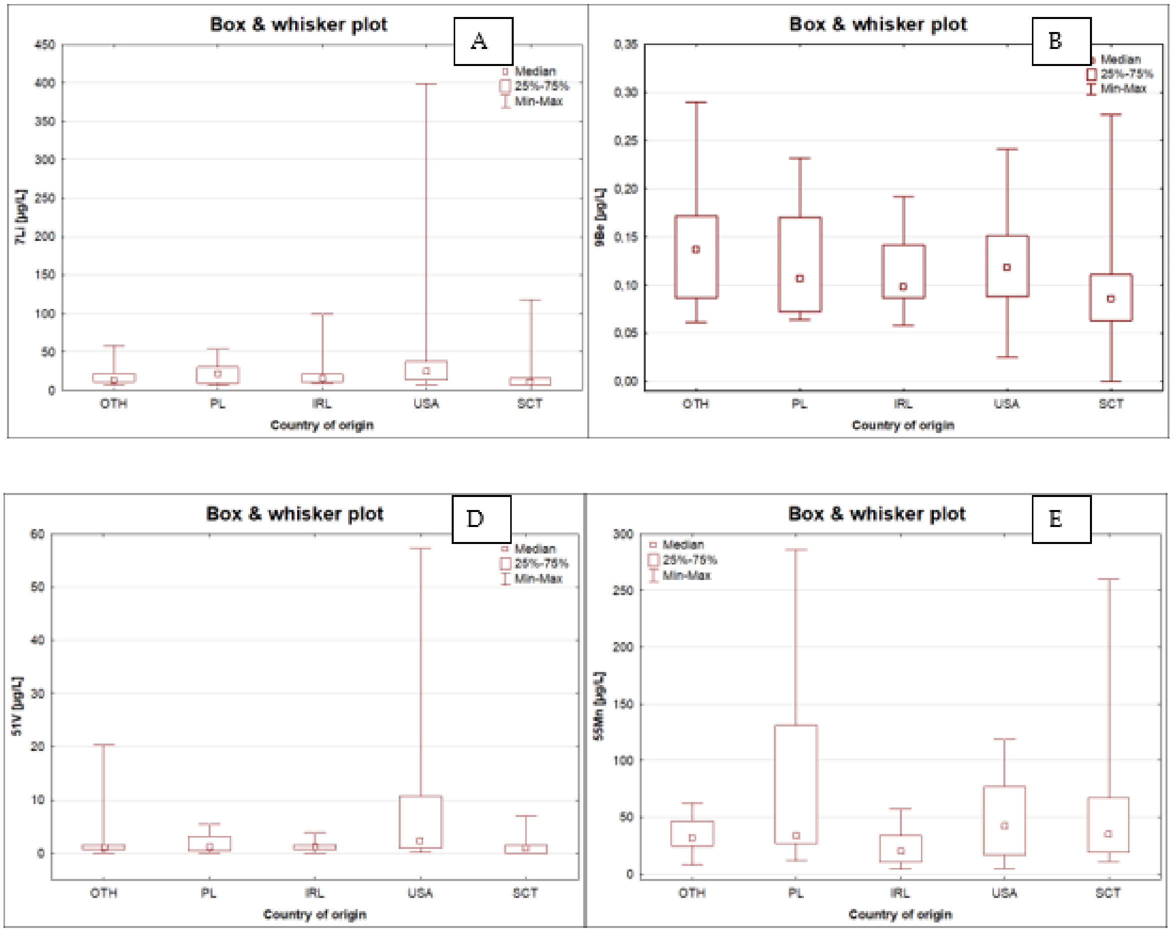
A
Box & whisker plot
Median
25%-75%
Min-Max
7Li [µg/L]
Country of origin
OTH
PL
IRL
USA
SCT
B
Box & whisker plot
9Be [µg/L]
D
Box & whisker plot
51V [µg/L]
E
Box & whisker plot
55Mn [µg/L]

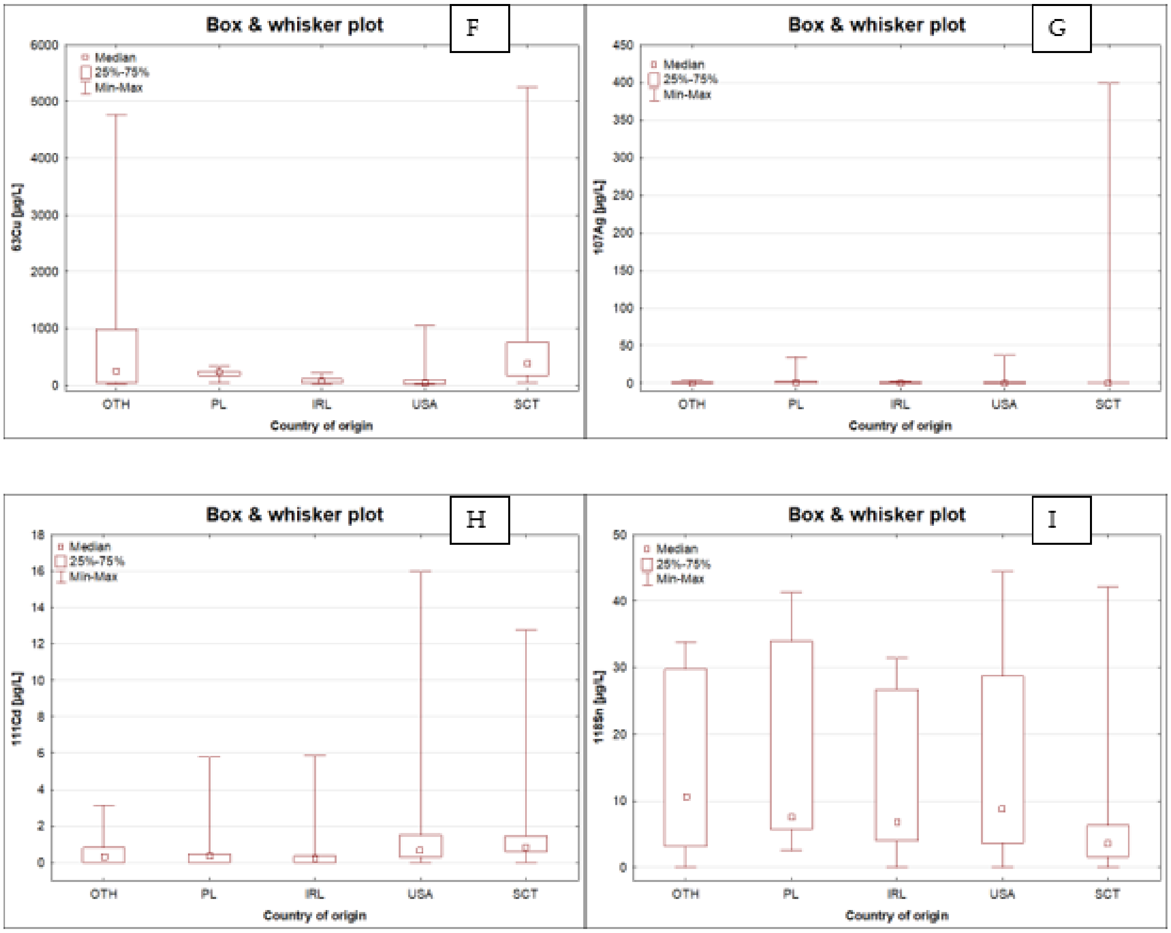
Box & whisker plot
F
Median
25%-75%
Min-Max
63Cu [µg/L]
0
1000
2000
3000
4000
5000
6000
OTH
PL
IRL
USA
SCT
Country of origin
Box & whisker plot
G
Median
25%-75%
Min-Max
107Ag [µg/L]
0
50
100
150
200
250
300
350
400
450
OTH
PL
IRL
USA
SCT
Country of origin
Box & whisker plot
H
Median
25%-75%
Min-Max
111Cd [µg/L]
0
2
4
6
8
10
12
14
16
18
OTH
PL
IRL
USA
SCT
Country of origin
Box & whisker plot
I
Median
25%-75%
Min-Max
118Sn [µg/L]
0
10
20
30
40
50
OTH
PL
IRL
USA
SCT
Country of origin

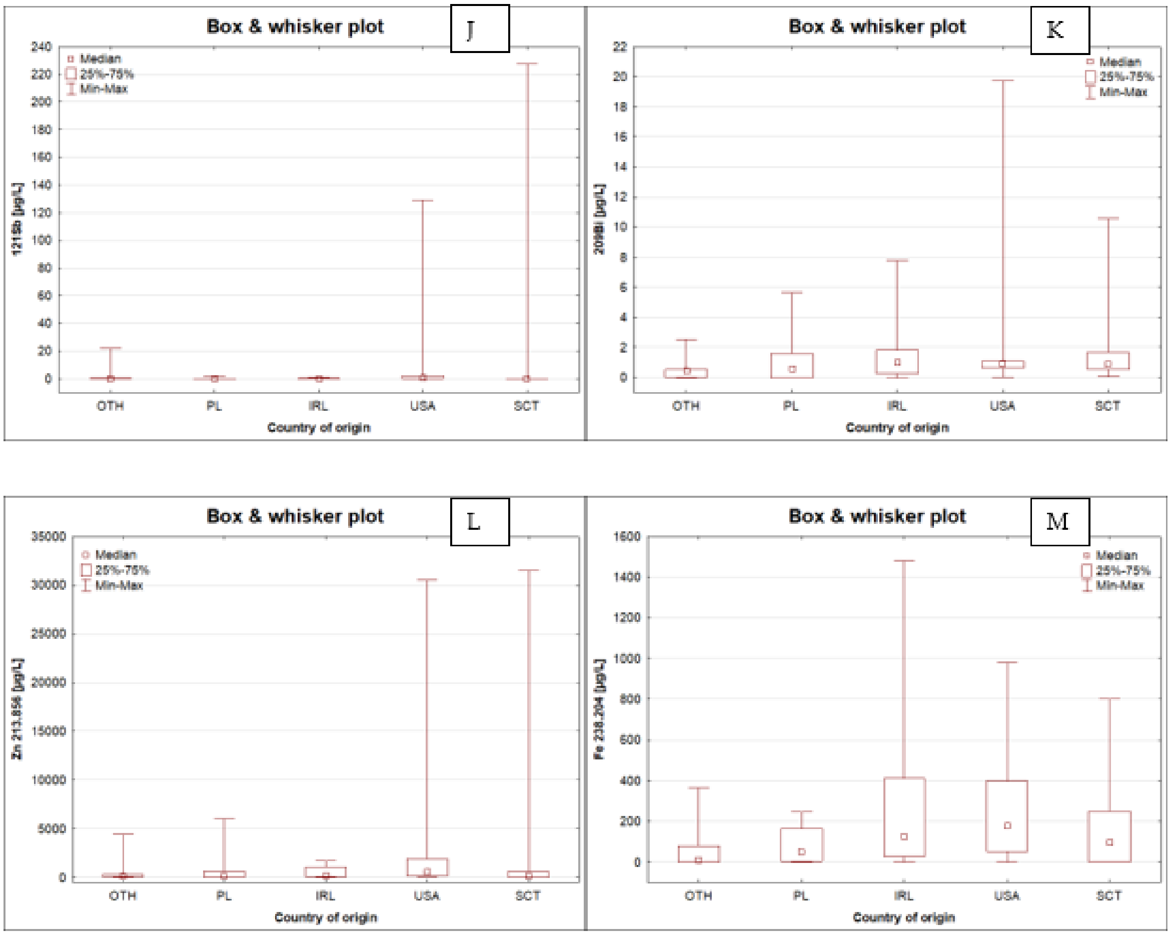
Box & whisker plot
J
Median
25%–75%
Min–Max
121Sb [µg/L]
OTH
PL
IRL
USA
SCT
Country of origin
Box & whisker plot
K
209Bi [µg/L]
Box & whisker plot
L
Zn 213.856 [µg/L]
Box & whisker plot
M
Fe 238.204 [µg/L]

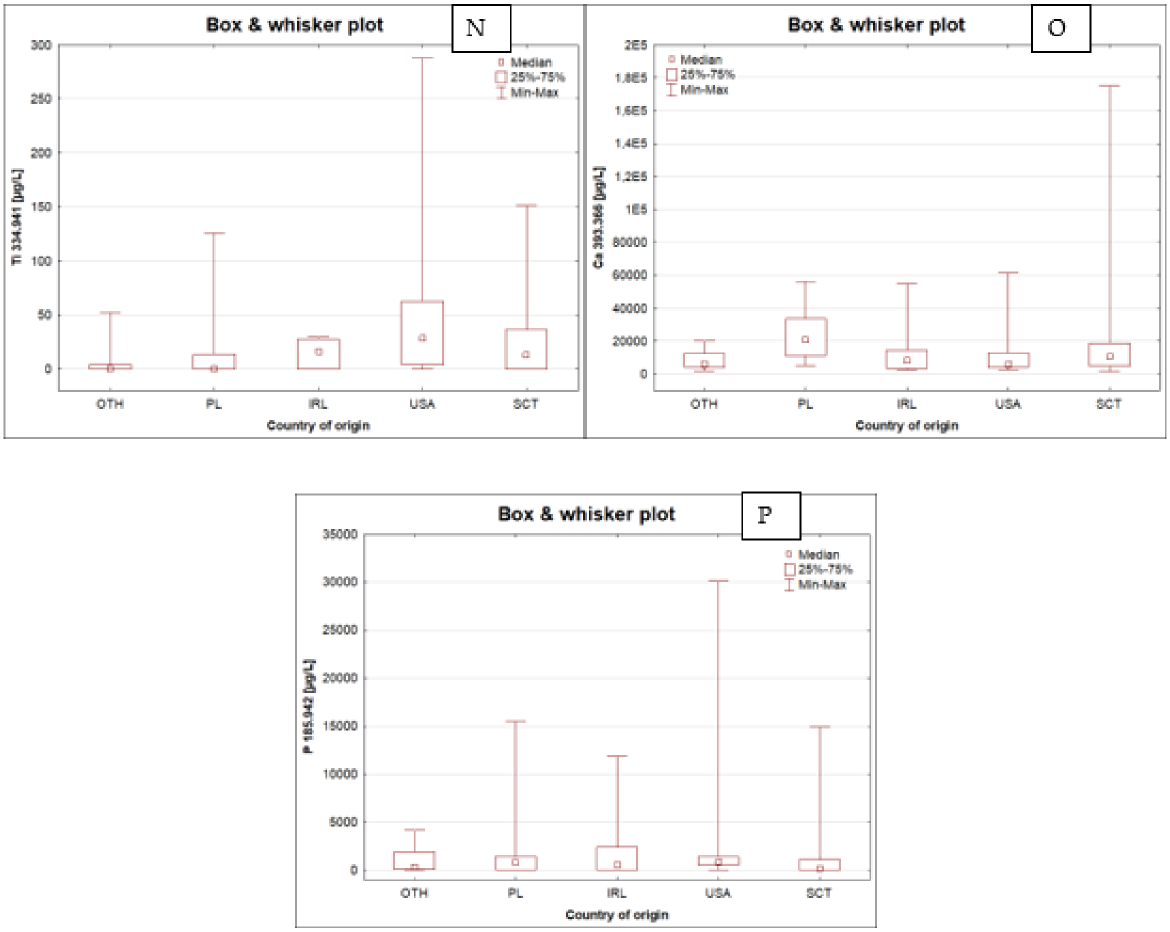

Figure S1. A-P. Box & whisker plots of selected elements (with statistically significant differences) in the measured whisky samples (n = 170) [µg/L].

Table S4. Contents of selected elements (with statistically significant differences) in the measured samples from USA division against the brand (n = 26) [μg/L].

Element	Brand code	n	Mean	Median	Min	Max	Std. Dev.
^{208}Pb	JB	6	12.02	10.35	8.975	19.01	3.783
	Oth	9	8.396	10.29	1.243	15.27	4.871
	JD	6	14.77	13.44	9.942	22.31	5.061
	Ole	3	2.590	2.650	0.283	4.836	2.277
	WilT	2	24.09	24.02	12.20	35.84	16.72
^{238}U	JB	6	0.479	0.428	0.306	0.704	0.1632
	Oth	9	0.224	0.190	0.055	0.593	0.1714
	JD	6	0.336	0.300	0.105	0.593	0.1713
	Ole	3	0.049	0.045	< LOQ	0.101	0.050
	WilT	2	0.097	0.097	0.040	0.155	0.081
^{55}Mn	JB	6	58.64	67.29	29.29	78.02	19.29
	Oth	9	66.43	77.23	16.61	118.5	32.29
	JD	6	18.39	16.64	4.809	35.70	13.06
	Ole	3	14.38	15.48	11.52	16.14	2.495
	WilT	2	79.51	79.51	64.28	94.74	21.54
^{95}Mo	JB	6	7.731	7.701	2.252	15.748	4.6420
	Oth	9	0.841	0.022	0.0010	2.946	1.2146
	JD	6	2.161	2.0577	0.0010	5.701	2.0880
	Ole	3	< LOQ	< LOQ	< LOQ	< LOQ	< LOQ
	WilT	2	0.751	0.751	< LOQ	1.502	1.061
Mg 279.553	JB	6	1409	1374	1004	1843	385.8
	Oth	9	1487	1413	912.9	1973	341.5
	JD	6	788.9	824.2	317.3	1194	298.5
	Ole	3	2469	2318	1246	3844	1306
	WilT	2	964.4	964.4	698.8	1230	375.7

Table S5. Contents of B in the measured samples from Ireland division against the brand (n = 15) [μg/L].

Brand code.	n	Mean	Median	Min	Max	Std. Dev.
Bus	7	3599	3975	925.6	5368	1865
Jam	3	8209	8148	8065	8396	172.2
Tul	2	4061	4061	2371	5752	2391
Oth	3	5801	6457	3193	7754	2350

Table S6. Contents of selected elements (with statistically significant differences) in the measured Scottish whisky (n = 106) [μg/L].

Elements	Type of Scotch whisky	n	Mean	Median	Min	Max	Std. Dev.
^{27}Al	B	56	129.3	138.4	< LOQ	262.4	72.12
	SM	50	92.19	77.39	< LOQ	225.3	61.88
^{51}V	B	56	0.681	0.281	< LOQ	7.060	1.098
	SM	50	1.530	1.330	< LOQ	5.700	1.240
^{52}Cr	B	56	173.2	111.4	53.83	666.1	135.0
	SM	50	62.59	66.69	10.70	108.6	27.69
^{55}Mn	B	56	24.34	21.90	11.28	63.35	10.66
	SM	50	77.90	71.14	16.85	260.1	47.44
^{63}Cu	B	56	284.3	187.9	48.55	2381	332.7
	SM	50	920.7	663.1	143.2	5252	817.8
^{205}Tl	B	56	0.130	0.046	< LOQ	2.580	0.357
	SM	50	0.09	0.02	< LOQ	1.500	0.230
Zn 213.856	B	56	417.9	97.62	< LOQ	4921	946.5
	SM	50	1790	195.8	< LOQ	31458	4917
Fe 238.204	B	56	70.51	6.065	< LOQ	473.0	109.4
	SM	50	248.7	223.3	< LOQ	802.3	217.7
Mg 279.553	B	56	874.0	812.3	208.5	3140	530.9
	SM	50	2005	1612	228.7	5833	1447
S 180.731	B	56	3931	3552	296.4	26663	3701
	SM	50	12680	9742	1610	69907	13167
P 185.942	B	56	339.8	109.3	< LOQ	5130	829.9
	SM	50	2476	974.9	< LOQ	14875	3459
K 766.490	B	56	9042	7379	< LOQ	23316	5415
	SM	50	29669	19596	< LOQ	149302	29246
Ti 334.941	B	56	18.80	6.601	< LOQ	149.0	29.57
	SM	50	29.37	14.70	< LOQ	151.1	35.79

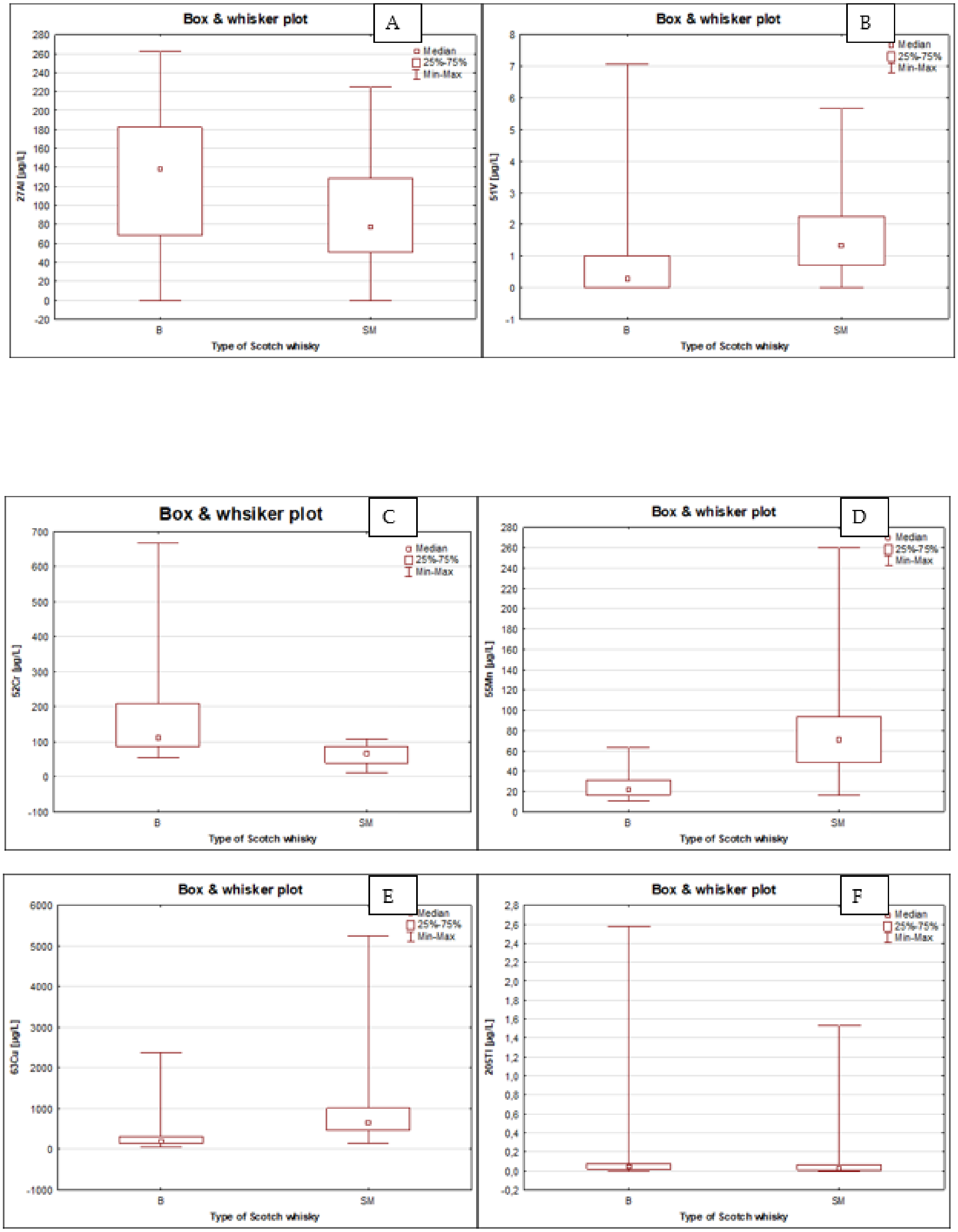
A
Box & whisker plot
Median
25%-75%
Min-Max
27Al [µg/L]
B
SM
Type of Scotch whisky
B
Box & whisker plot
51V [µg/L]
C
Box & whsiker plot
52Cr [µg/L]
D
Box & whisker plot
55Mn [µg/L]
E
Box & whisker plot
63Cu [µg/L]
F
Box & whisker plot
205Tl [µg/L]

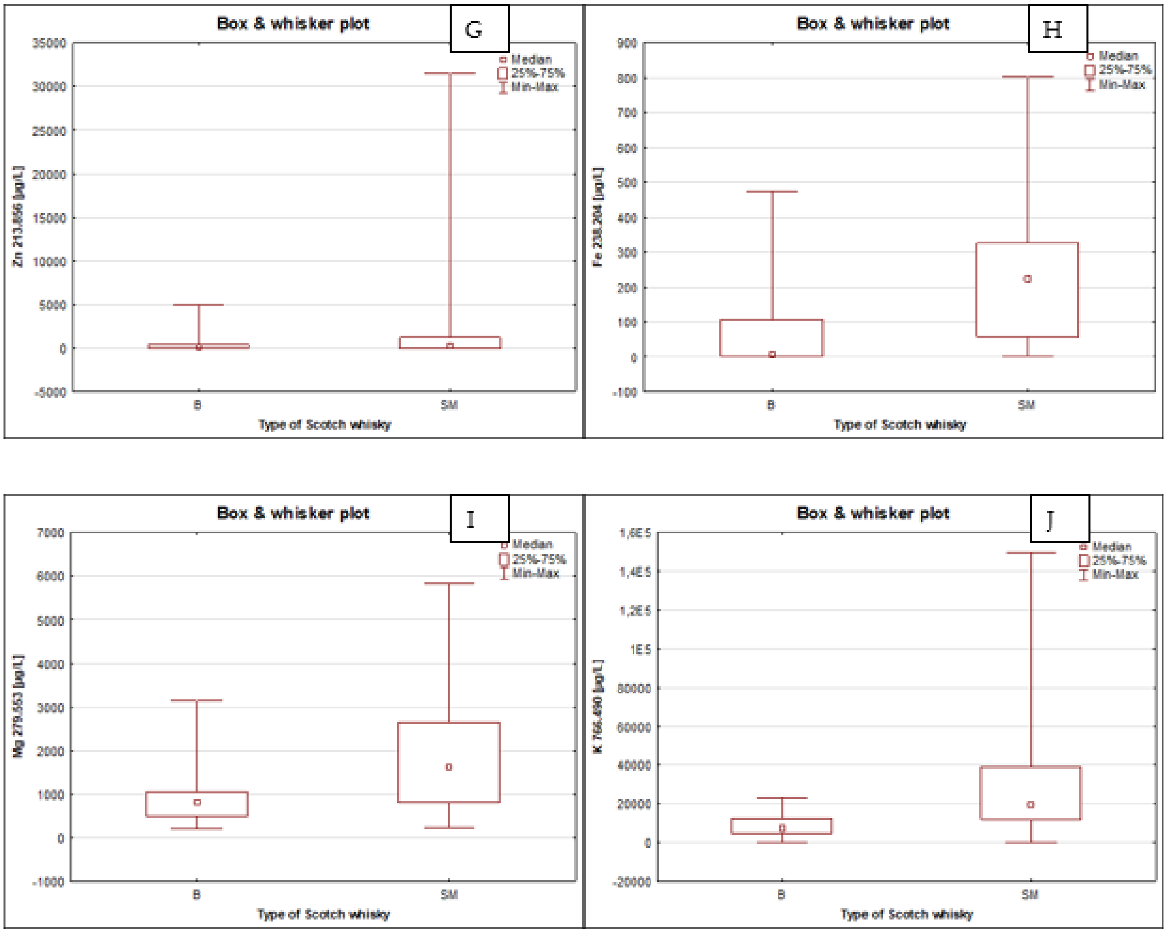
Box & whisker plot
G
Median
25%-75%
Min-Max
Zn 213.856 [µg/L]
Type of Scotch whisky
B
SM
Box & whisker plot
H
Fe 238.204 [µg/L]
Box & whisker plot
I
Mg 279.553 [µg/L]
Box & whisker plot
J
K 766.490 [µg/L]

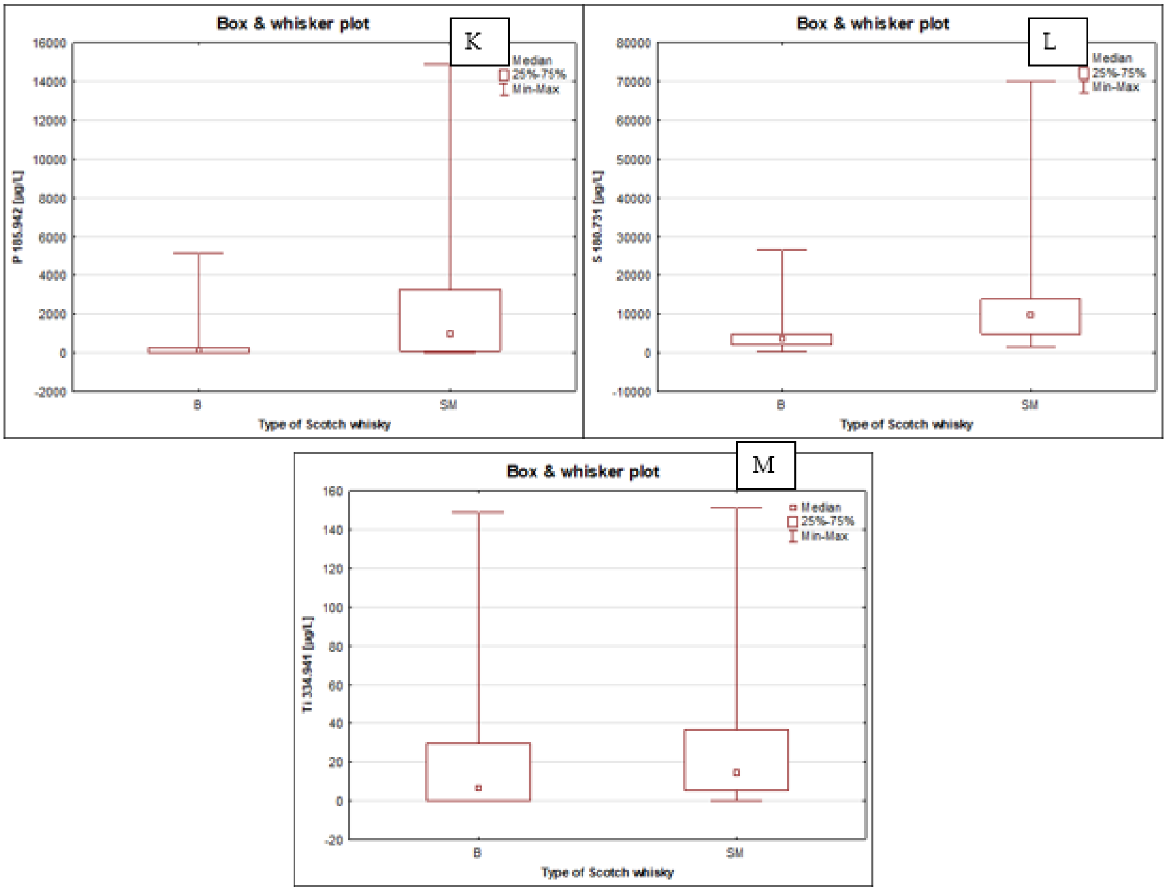

Figure S2. A-K. Box & whisker plots of selected elements (with statistically significant differences) in the measured Scottish whisky (n = 106) [µg/L].

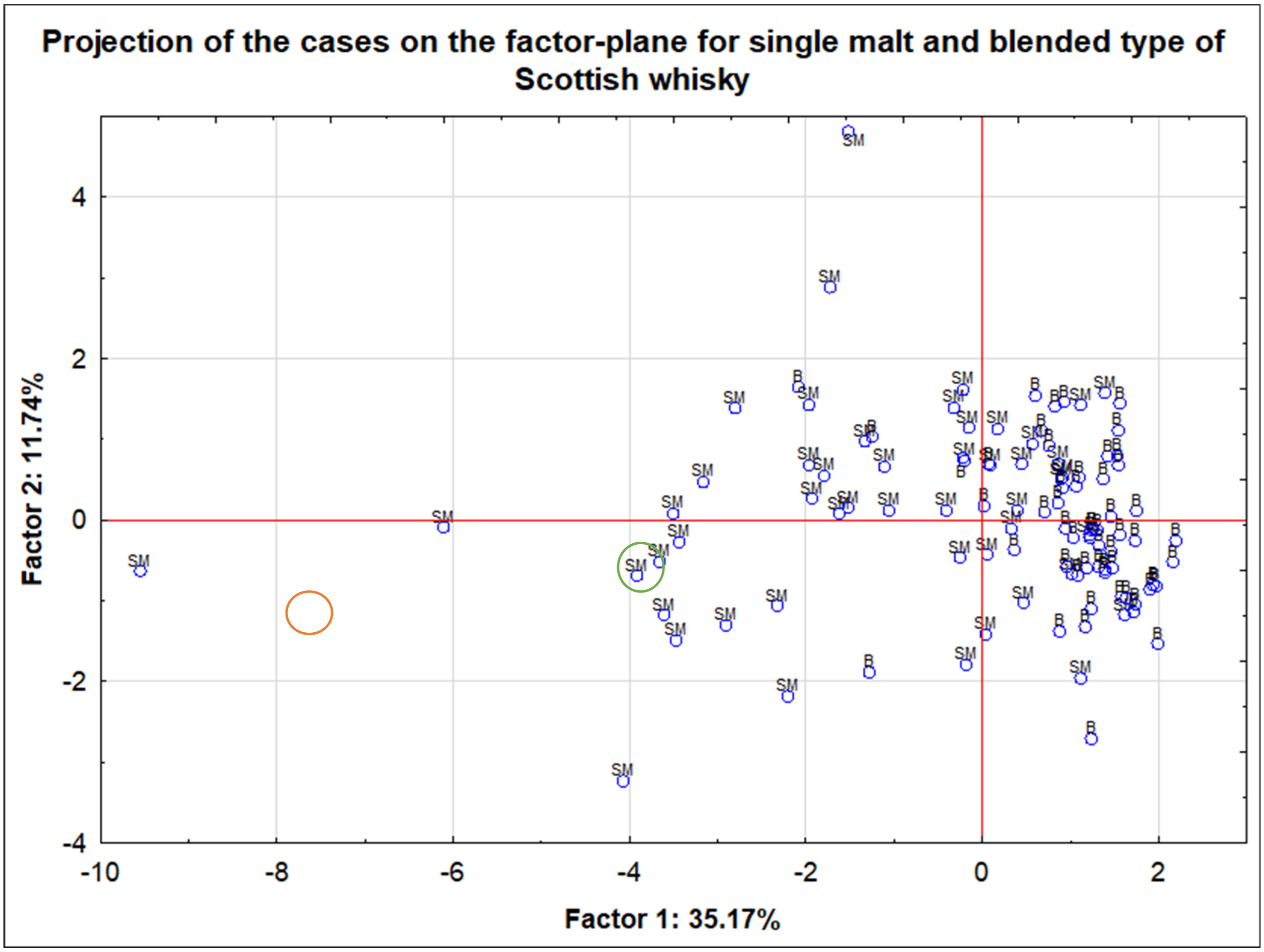

Figure S3. Projection of the cases on the factor-plane for 106 samples from Scotland according to their type (single malt (SM) and blended (B)).

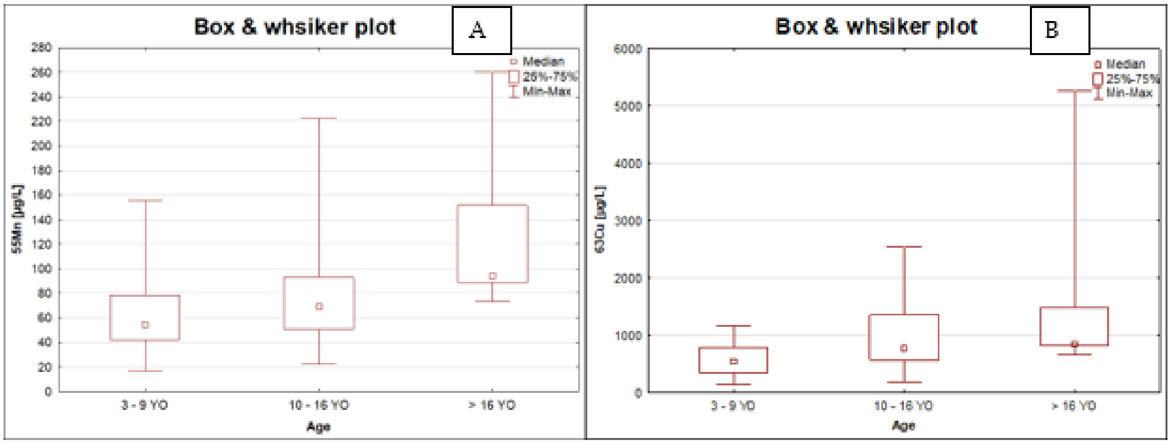

Figure S4. A-B. Box & whisker plots of selected elements (with statistically significant differences) in the measured Scottish single malt whisky (n = 50) [µg/L].

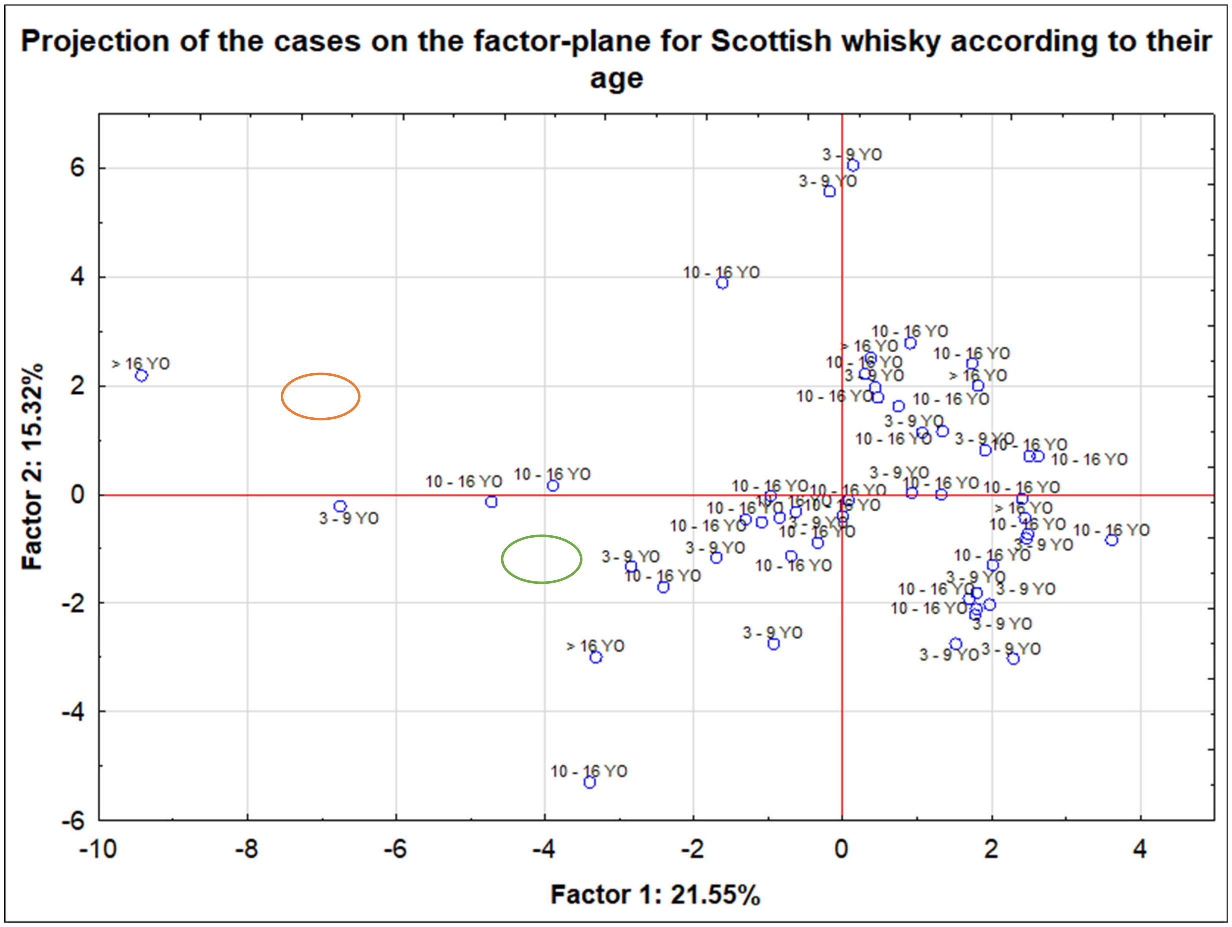

Figure S5. Projection of the cases on the factor-plane for 50 samples of single malt whisky from Scotland.
